# Challenges in the Diagnosis and Treatment of *Mycobacterium abscessus* Infections

**DOI:** 10.1590/0037-8682-0354-2025

**Published:** 2026-02-16

**Authors:** Ana Júlia Reis, Michael Andrés Abril Gómez, Mariana Quaresma de Souza, Júlia Silveira Vianna, Ivy Bastos Ramis, Pedro Eduardo Almeida da Silva

**Affiliations:** 1 Universidade Federal do Rio Grande, Faculdade de Medicina, Rio Grande, RS, Brasil.; 2 Rede TB (Rede Brasileira de Pesquisas em Tuberculose). Rio de Janeiro, RJ, Brasil.

**Keywords:** Antibiotic resistance, Diagnosis, Mycobacterium abscessus, Non-tuberculous mycobacteria, Pulmonary infections

## Abstract

The *Mycobacterium abscessus* complex (MABC) includes non-tuberculous mycobacteria that are widely distributed and clinically significant. Similar to tuberculosis, MABC can lead to skin and soft tissue infections and pulmonary diseases. These infections frequently occur in outbreaks, particularly among immunocompromised patients or those with preexisting pulmonary conditions. This review examines the recent progress in essential areas that define these infections as a significant challenge in medical practice, specifically the diagnostic modalities, antibiotic treatment options, and resistance of MABC to antibiotics and biocides.

## INTRODUCTION

Mycobacterial infections present a significant challenge to public health, including infections caused by non-tuberculous mycobacteria (NTM)^1^ ^,^ ^2^. Among NTM, the *Mycobacterium abscessus* complex (MABC) is one of the most pathogenic rapidly growing mycobacteria^3^ ^,^ ^4^.

**FIGURE 1:**
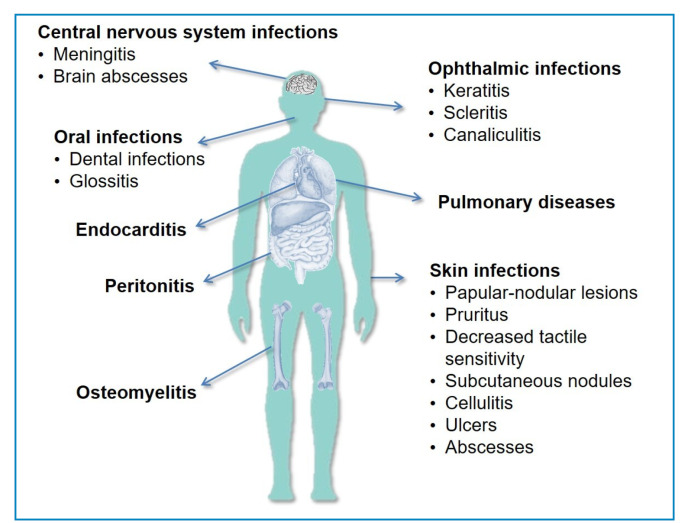
Main clinical manifestations caused by the *Mycobacterium abscessus* complex.

MABC infections often present as chronic lung diseases but can also affect the skin, soft tissues, and bones. Disseminated infections are common among immunocompromised individuals (Figure 1)^5^ ^-^ ^7^, such as patients with cystic fibrosis or those undergoing cancer treatment. Furthermore, MABC isolates are responsible for a variety of infections, particularly in immunocompromised patients (e.g.*,* human immunodeficiency virus-infected individuals with low CD4+ T-cell counts), resulting in elevated mortality and morbidity rates^6^ ^,^ ^7^. In addition, MABC infections may coincide with other microbial infections, including those caused by various mycobacterial species^8^ ^-^ ^10^.

**FIGURE 2:**
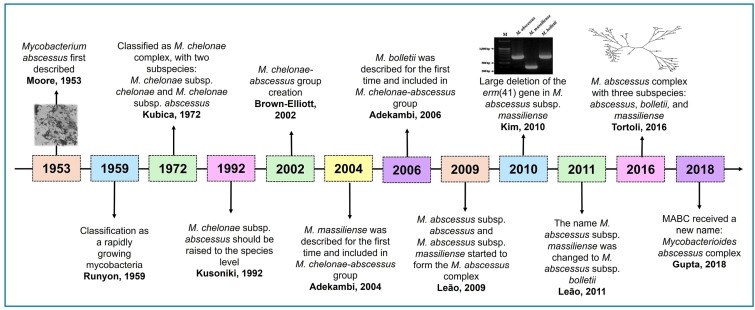
Classification of the MABC over a 65-year timeline. **MABC:** *Mycobacterium abscessus* complex.

The taxonomic classification of MABC has undergone several revisions (Figure 2). It currently comprises three subspecies: *M. abscessus* subsp. *abscessus*, *M. abscessus* subsp. *bolletii*, and *M. abscessus* subsp. *massiliense*. These subspecies exhibit distinct genotypic and phenotypic characteristics, including different antibiotic susceptibility profiles^11^. Accurate identification at the subspecies level is crucial because *M. abscessus* subsp. *abscessus* and *M. abscessus* subsp. *bolletii* possess functional *erm*(41) genes, leading to inducible resistance to macrolides, such as clarithromycin (CLA). In contrast, *M. abscessus* subsp. *massiliense* lacks the functional *erm*(41) gene, making it more susceptible to macrolide treatment^12^ ^,^ ^13^. The intrinsic resistance of MABC to many antibiotics complicates the treatment strategies that can be applied for MABC-related infections.

MABC infections are prevalent worldwide. Due to the ability of MABC to survive in various environments and adhere to diverse surfaces, leading to biofilm formation, these infections are commonly acquired in natural or healthcare settings^2^ ^,^ ^5^ ^,^ ^14^. Therefore, significant concern exists regarding outbreaks^15^ ^,^ ^16^, particularly for procedures involving parenteral drugs, surgical instruments, or contaminated solutions^15^ ^-^ ^17^. 

To enhance our understanding of infections caused by MABC, in this integrative review, we synthesize and critically analyze recent MABC-related advances, focusing on key aspects that contribute to the recognition of MABC as a major challenge in clinical practice, including the current diagnostic approaches, available antimicrobial therapies, and mechanisms of resistance to antibiotics and biocides.

## METHODS

For this integrative literature review, the following methodological steps were employed: (1) formulation of the research question: What recent advances related to the MABC in diagnosis, treatment, and antimicrobial resistance have contributed to its recognition as a major challenge in clinical practice?, (2) definition of inclusion and exclusion criteria, (3) systematic literature search, (4) study selection, (5) data extraction and analysis, and (6) synthesis and presentation of results.

The literature search was performed in the PubMed/MEDLINE, Scopus, Web of Science, SciELO, and LILACS databases using descriptors combined with the Boolean operators “AND” and “OR.” This review included full-text studies published in English, Portuguese, and Spanish. Duplicate studies, reports with insufficient data, conference abstracts, Letters to the Editor, and any such items that did not address the research question were excluded.

The selection process was conducted in two stages: initial screening of titles and abstracts, followed by full-text reading of eligible articles. This study analyzed the collected data in a descriptive and interpretative manner, allowing the identification of trends, knowledge gaps, and challenges related to the management of MABC-related infections. This review synthesizes the results of our literature search and organizes them into the three main thematic categories of diagnosis, treatment, and antibiotic and biocide resistance.

## DIAGNOSIS

The diagnosis of MABC-related lung disease involves the integration of clinical assessment, medical imaging, and microbiological findings (Table 1). The American Thoracic Society and Infectious Diseases Society of America guidelines for diagnosing pulmonary NTM infections mandate a minimum of two positive sputum cultures collected on different occasions. Given the ubiquitous presence of NTM in the environment, meticulous assessment is necessary to detect MABC in sputum. However, a single positive culture from a lower respiratory tract specimen, such as a bronchial lavage or washing, is satisfactory for diagnosis^18^.

**TABLE 1:** American Thoracic Society Criteria for non-tuberculous mycobacteria lung disease diagnosis.

Findings category^a^	Diagnosis criteria
Clinical	Symptoms as cough (most prevalent symptom), sputum production, fatigue, malaise, dyspnea, fever, hemoptysis, chest pain, and weight loss;
	**AND**
	Appropriate exclusion of other diagnoses, such as tuberculosis and lung malignancy.
Medical imaging	Chest radiography showing nodular or cavitary opacities;
	**OR**
	High-resolution computed tomography showing multifocal bronchiectasis with multiple small nodules.
Microbiological	For sputum, positive culture results from at least two separate expectorated samples. Repeated sputum acid-fast bacilli smears and cultures are recommended if the results from the first sputum are not enough to diagnosis;
	**OR**
	For bronchial wash/lavage, positive culture result from at least one sample; OR For a biopsy, positive culture is associated with mycobacterial histopathologic features (granulomatous inflammation or acid-fast bacilli). In the absence of positive culture, besides mycobacterial histopathologic features, it is necessary to be associated with one or more sputum or bronchial washings that are culture positive.

^a^Table adapted from Griffith et al, 2007^5^.

As a diagnosis based solely on clinical and imaging characteristics is not sufficient for initiating treatment^5^ ^,^ ^19^, we address standard laboratory diagnostic methods. MABC infections can be diagnosed using several phenotypic tools, such as microscopy, culture, and biochemical tests, as well as via genotypic (molecular) techniques, which enable the precise identification of MABC species^7^ ^,^ ^20^ ^,^ ^21^.

### Phenotypic diagnosis

Smear microscopy is a fundamental diagnostic tool for mycobacterial infections and is routinely used in most laboratories. This technique is simple, fast, and cost-effective. However, its sensitivity is limited, and it cannot distinguish between species of acid-fast bacilli^22^.

Although mycobacterial culture requires higher biosafety standards and longer processing times, its specificity and sensitivity are superior to those of microscopy^23^ ^,^ ^24^. Cultures are typically conducted on solid media, such as Ogawa-Kudoh and Löwenstein-Jensen, or in liquid media, such as Middlebrook 7H9, often using the semi-automated BACTEC MGIT^®^ system (BD, USA). MABC bacterial growth is generally detectable within approximately 7 days on solid media and as early as 5 days in liquid culture systems^5^ ^,^ ^25^.

Phenotypic characteristics, including growth rate and pigment production, offer an initial method for identifying MABC in bacterial isolates. The literature describes MABC species based on their rapid growth and lack of pigment production. Biochemical assays serve as alternative diagnostic tools in clinical laboratories with limited resources or a lack of molecular identification capabilities. MABC tests typically show positive results for arylsulfatase production, growth on MacConkey agar (supplemented with crystal violet and 5% NaCl), and citrate utilization^26^. However, combining phenotypic methods with genotypic (molecular) diagnostics or mass spectrometry-based techniques is highly recommended.

### Genotypic (molecular) diagnosis

***Line probe assay:*** The line probe assay (LPA) relies on reverse hybridization and uses DNA probes attached to a nitrocellulose membrane that hybridizes with specific regions of mycobacterial species^27^. A commonly employed LPA assay involves amplification of the 23S rRNA gene region, which can be used to identify up to 27 NTM species in culture. An alternative iteration of this assay can assist in directly identifying 20 NTM species in patient specimens^28^.

Another method for the rapid identification of mycobacteria involves an oligochromatographic assay that relies on amplifying the 16S rRNA fragment and the spacer region of the 16S-23S rDNA gene. This assay combines double reverse hybridization with probes linked to colloidal gold. The LPA can be conducted on bacterial cultures and typically takes approximately 3 h, including DNA extraction, to yield results^29^ ^-^ ^31^.

***Polymerase chain reaction and gene sequencing:*** In recent decades, nucleic acid amplification-based techniques, such as polymerase chain reaction (PCR) and DNA sequencing, have become essential tools for clinical mycobacteriology laboratories. These genotypic methods are faster and more accurate than the phenotypic methods^22^ ^,^ ^32^ ^,^ ^33^.

Restriction enzyme pattern analysis (PRA) is a common method for identifying NTM species. This technique relies on PCR amplification of a 439-bp fragment of the *hsp65* gene, followed by digestion of the amplified product using *BstE*II and *Hae*III restriction enzymes^34^. PRA is a relatively fast and inexpensive method. However, certain species exhibit indistinguishable or highly similar patterns, which complicates species differentiation. Currently, no established standards exist for differentiating species using PRA. Hence, additional tests are required to validate the results^35^ ^,^ ^36^.

MABC subspecies can be distinguished by identifying the *erm*(41) and *mass* genes through conventional PCR amplification. The *erm*(41) gene is 673 bp in *M. abscessus* subsp. *abscessus* and *M. abscessus* subsp. *bolletii* isolates and 397 bp in *M. abscessus* subsp. *massiliense* isolates^37^. In contrast, the *mass_3210* gene is 310 bp in *M. abscessus* subsp. *abscessus* and *M. abscessus* subsp. *bolletii* isolates*,* and 1,145 bp in *M. abscessus* subsp. *massiliense* isolates^38^.

Moreover, utilizing DNA sequencing to analyze additional genes such as the *16S* rRNA, *rpoB*, *hsp65*, *secA*, *sodA*, and ITS regions provides sensitive and accurate MABC identification^39^ ^-^ ^41^. However, sequencing a single gene lacks sufficient discriminatory power due to horizontal gene transfer among subspecies^42^. Although DNA sequencing is a highly sensitive technique for diagnosing MABC, numerous clinical laboratories lack the resources for performing routine sequencing^43^.

Considering the differences among MABC subspecies, especially their antibiotic susceptibilities, methods that can identify these subspecies are essential for the accurate treatment of these infections.

***Matrix-assisted laser desorption/ionization-time of flight mass spectrometry:*** Matrix-assisted laser desorption/ionization-time of flight mass spectrometry (MALDI-TOF MS) is a valuable tool for identifying MABC in clinical laboratories^44^. Although this technique is highly accurate in distinguishing NTM species, differentiating among MABC subspecies is challenging because of their genetic^45^ **.** Recent research has shown that combining machine learning algorithms with MALDI-TOF MS protein spectra can greatly improve subspecies-level identification^46^.

In conclusion, to diagnose MABC infections, a combination of clinical assessments, imaging examinations, and microbiological procedures is required to ensure diagnostic accuracy. Although phenotypic tools are crucial, genotypic (molecular) methods have significantly improved the diagnostic accuracy^47^. The advent of MALDI-TOF MS and machine learning technologies has improved the prompt identification of MABC subspecies and has facilitated appropriate treatment decisions. Considering the clinical significance of accurate MABC infection classification, incorporating advanced diagnostic strategies is essential for enhancing patient care and treatment results.

## TREATMENT

MABC infections necessitate extended treatment (>12 months) and the utilization of a minimum of three antibiotics guided by *in vitro* susceptibility^19^. Prolonged multitherapy increases the risk of adverse effects or toxicity and poses significant treatment adherence challenges^48^ ^,^ ^49^. Macrolides, including CLA, azithromycin, and erythromycin, are the primary antibiotics for treating MABC infections and are often combined with parenteral agents such as amikacin, imipenem, cefoxitin, or tigecycline. Additionally, oral antibiotics, including linezolid, trimethoprim-sulfamethoxazole, clofazimine, certain quinolones (ciprofloxacin and moxifloxacin), and tetracycline derivatives (doxycycline) may be administered^19^. Unfortunately, multidrug treatment may be inadequate for certain MABC infection sites, such as the lungs and skin. In these cases, the concomitant management of lesions includes surgical excision of necrotic tissue or drainage of abscesses^50^.

Although multidrug therapy is effective in most cases, high antibiotic resistance rates, particularly CLA resistance, are directly associated with therapeutic failure^51^ ^-^ ^53^. Contrastingly, low *in vitro* resistance rates (<4%) to amikacin have been observed in *M. abscessus* subsp. *abscessus* and *M. abscessus* subsp. *massiliense* isolates^54^. However, amikacin is an intravenous antibiotic with numerous adverse effects that hamper its use in clinical practice. A previous study showed that amikacin treatment had to be discontinued or adjusted in 51% of patients^48^. As an alternative, inhaled amikacin has been introduced to treat MABC infections; the inhaled form reduces the adverse effects of amikacin and increases its antimicrobial efficacy^49^.

A study assessing the therapeutic response in patients with MABC infection revealed that the percentage of MABC-infected patients who did not respond to treatment was nearly 12 times greater than that of other NTM species-infected patients^55^. Furthermore, the treatment response of patients with lung infections induced by *M. abscessus* subsp. *abscessus* was poorer than that of patients with multidrug-resistant tuberculosis (TB), akin to that of patients with extensively resistant TB^56^.

Therefore, new therapeutic alternatives are urgently required to enhance the efficacy of MABC treatment. Studies have shown the effectiveness of novel antibiotics such as rifabutin, clofazimine, bedaquiline, and tedizolid against MABC^57^ ^-^ ^59^. Specifically, rifabutin has demonstrated potent bactericidal activity against isolates from various MABC subspecies, including those resistant to CLA^57^ ^,^ ^60^. Even though these antibiotics are potential treatment options for MABC, several studies have highlighted their limitations. A recent study investigating the mechanisms of action of clofazimine and bedaquiline revealed cross-resistance to these antibiotics^61^. Furthermore, evaluation of *in vitro* bedaquiline susceptibility in MABC samples indicated a resistance rate of approximately 20%^62^.

Numerous clinical trials worldwide have assessed the efficacy of different compounds for managing MABC infections^63^. A phase III clinical cohort study examined various antibiotic combinations, including amikacin, tigecycline, imipenem, cefoxitin, azithromycin, CLA, clofazimine, ethambutol, linezolid, cotrimoxazole, doxycycline, moxifloxacin, bedaquiline, and rifabutin, in patients with lung disease caused by MABC subspecies to enhance treatment outcomes and reduce toxicity^63^. Moreover, a preclinical mouse model infected with MABC demonstrated that a combination of clofazimine and bedaquiline was the most effective for reducing the MABC burden in organs such as the lungs, spleen, and liver^64^. Another study revealed that a novel therapy employing apoptotic body-like liposomes (ABL/PI5P) enhanced bacterial clearance and reduced inflammation by boosting the macrophage response. When combined with amikacin, this approach improved infection control; thus, it is a promising strategy for treating infections caused by multidrug-resistant *M. abscessus* ^65^.

## ANTIBIOTIC AND BIOCIDE RESISTANCE

Drug resistance is a highly significant phenomenon in MABC infections because of its correlation with elevated treatment failure^6^. Another crucial aspect of MABC infection treatment is the varying susceptibility profiles of MABC subspecies, particularly to CLA.

**FIGURE 3:**
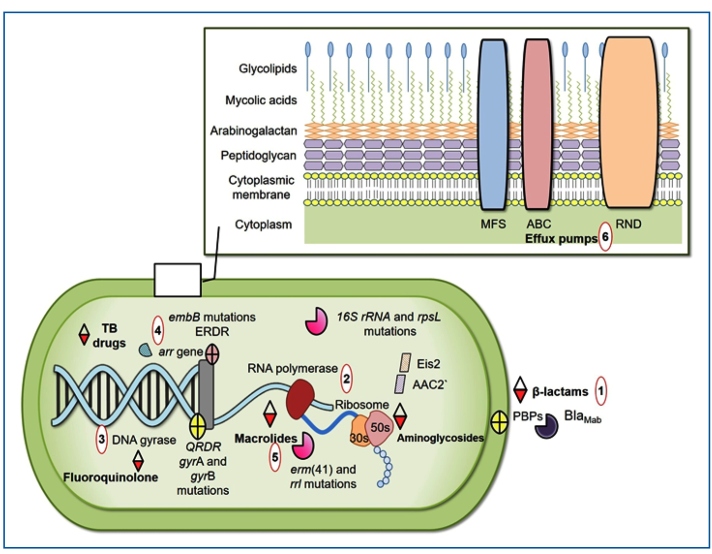
Mechanisms of antibiotic resistance in the MABC. (1) β-lactam inactivation due to hydrolysis by the Bla^Mab^ enzyme. (2) Aminoglycoside resistance related to enzymatic modifications (AAC2' and Eis2) or mutations in the 16S rRNA and *rpsL* genes. (3) Fluoroquinolone resistance determined by mutations in QRDRs (quinolone resistance-determining regions within the *gyrA* and *gyrB* subunits of DNA gyrase). (4) Resistance to anti-TB drugs (ethambutol, rifampin, or isoniazid) associated with mutations in the conserved ERDR (ethambutol resistance-determining region) of the *embB* gene, the presence of an *arr* (ADP-ribosyltransferase) gene, and intracellular enzymes that acetylate and inactivate antibiotics. (5) Macrolide resistance conferred by point mutations in *rrl* (23S rRNA) and the presence of *erm*(41), which induces resistance to these antibiotics. (6) Efflux pumps mechanism that can export different antibiotics out of the cell, leading to prolonged bacterial exposure to subinhibitory antibiotic concentrations. **MFS:** major facilitator superfamily; **ABC:** ATP-binding cassette; **RND:** resistance nodulation division; **TB:** tuberculosis; **PBPs:** Penicillin-Binding Proteins.

The MABC exhibits high rates of intrinsic and acquired resistance to multiple antibiotics^60^ ^,^ ^66^ ^-^ ^72^. Regarding intrinsic resistance, the barriers include physical (size exclusion) and chemical (hydrophobic) factors, drug export mechanisms, enzymes that can modify drugs or target enzymes, and genetic variations in the target genes (Figure 3)^73^ ^,^ ^74^. Furthermore, some MABC molecular bases may confer both intrinsic and acquired resistance to several antibiotic classes, including anti-tuberculosis drugs, fluoroquinolones, aminoglycosides, beta-lactams, and macrolides^66^ ^,^ ^68^ ^-^ ^70^ ^,^ ^75^. Herein, we emphasize the genotypes associated with CLA resistance, as CLA is the most vital antibiotic for treating MABC infections.

Mutations in conserved genes are commonly linked to susceptibility or antibiotic resistance genotypes^66^ ^,^ ^68^ ^-^ ^70^. In this context, MABC exhibits resistance to anti-TB drugs, such as rifampicin and isoniazid^76^. Intrinsic resistance to rifampicin is associated with the presence of the *arr* (ADP-ribosyltransferase) gene^70^. Intracellular enzymes that acetylate and inactivate antibiotics confer intrinsic resistance to isoniazid^67^. Among these, MABC subspecies demonstrate high levels of acquired resistance to ethambutol, often due to mutations in the conserved ethambutol resistance-determining regions of *embB* ^66^.

Despite its intrinsic resistance to rifampicin, studies have shown promising activity of rifabutin, a close derivative of rifampicin, against MABC^57^ ^,^ ^60^. The activity of rifabutin against MABC isolates can be explained by the fact that unlike rifampicin, rifabutin is not inactivated by ADP-ribosyltransferase^77^ ^,^ ^78^.

A prior study revealed that an alanine residue at position 83 of *gyrA* (Ala-83) contributes to the intrinsic fluoroquinolone resistance of MABC. Acquired resistance is associated with mutations in *gyrB* at positions 426, 447, and 464, which encode asparagine (Asp-426), arginine (Arg-447), and asparagine (Asn-464), respectively^68^.

The primary mechanism of aminoglycoside resistance in MABC involves mutations in the *16S* rRNA and *rpsL* genes, which account for approximately 90% of acquired aminoglycoside resistance cases. Mutations, such as the adenine to guanine substitution at position 1408 (A1408G) within the *16S* rRNA gene, are linked to high acquired resistance levels to kanamycin, amikacin, gentamicin, and tobramycin in clinical MABC isolates^69^. In addition to these mutations, modifying enzymes such as AAC(2') and Eis contribute to intrinsic aminoglycoside resistance. It has been proposed that deletion of the *aac(2’)* and *eis2* genes, which encode the AAC(2') and Eis2 enzymes, respectively, is associated with increased susceptibility of MABC isolates to aminoglycosides^75^.

MABC isolates also exhibit inherent resistance to β-lactams. The literature identifies the β-lactamase Bla_Mab_ as the primary determinant of β-lactam resistance in MABC. One approach to broaden the range of β-lactam antibiotics is through the utilization of β-lactamase inhibitors, including avibactam, relebactam, and vaborbactam^71^ ^,^ ^72^ ^,^ ^79^.

***Molecular bases related to macrolide resistance:*** Macrolides constitute an antibiotic class distinguished by a structure that includes amino sugars and/or neutral sugar components connected to a lactone ring, producing macrolides with 12-, 14-, 15-, or 16-membered rings via glycosidic linkages. Their mechanism of action involves binding to the 50S ribosomal subunit in bacteria, inhibiting protein synthesis^73^ ^,^ ^80^.

MABC has two primary predictive genotypes for CLA resistance: *erm*(41), which confers induced resistance^81^, and a point mutation in *rrl* (*23S* rRNA), which confers acquired resistance^82^. With a highly conserved genome, a methylase encoded by *erm*(41) inhibits macrolides from binding to the active site of the 50S ribosomal subunit. The *erm*(41) gene leads to isolates that are initially susceptible to macrolides in *in vitro* tests; however, prolonged exposure induces resistance to CLA^53^ ^,^ ^83^.

Patients infected with *M. abscessus* subsp. *massiliense* typically exhibit a positive response to macrolide treatment because of *erm*(41) inactivity, which is characterized by a 2-276 bp deletion. For this subspecies, previous studies showed no induction of CLA resistance. In general, *M. abscessus* subsp. *abscessus* presents induced resistance to CLA due to the presence of an active *erm*(41) gene (sequevar T28). However, some strains show a thymine-to-cytosine polymorphism at the 28^th^ nucleotide of *erm*(41), which renders the gene inactive (sequevar C28). Patients infected with *M. abscessus* subsp. *bolletii* isolates do not respond well to treatment because they have the active *erm*(41) gene form (sequevar T28)^13^ ^,^ ^53^ ^,^ ^81^.

Expression of the *erm*(41) gene provides MABC with inherent resistance to macrolides such as CLA, erythromycin, and azithromycin^53^ ^,^ ^81^ ^,^ ^83^. Identification of *erm*(41) and its association with inducible CLA resistance has elucidated the ineffectiveness of macrolides in treating MABC infections and aided in interpreting the inaccurate outcomes of susceptibility tests^53^.

Another gene associated with CLA resistance is *rrl*, which is responsible for encoding the peptidyl transferase domain of the *23S* rRNA gene^53^ ^,^ ^82^. Point mutations at positions A2058 and A2059 in these regions-involving adenine to cytosine, adenine to thymine, or adenine to guanine substitutions-have been linked to CLA resistance^81^ ^,^ ^82^. Consequently, clinical instances of treatment failure due to mutations in *rrl* are frequently encountered^81^.

Although the T28 sequevar is associated with induced resistance to CLA, a study conducted on MABC strains from Brazil and Portugal revealed that only three strains exhibited induced resistance. These included two from *M. abscessus* subsp. *abscessus* and one from *M. abscessus* subsp. *bolletii* out of ten strains tested with the T28 sequevar. These findings imply that the T28 polymorphism in *erm*(41) may not be the sole factor contributing to the CLA-induced resistance in MABC strains^84^.

In MABC*,* the mycobacterial transcriptional regulator WhiB7 regulates stress responses and antibiotic resistance by regulating the expression of *erm*(41) and *eis*2, thereby enhancing resistance. Deletion of *whiB7* increases the susceptibility to various antibiotics, except rifampin and isoniazid^73^.

***Efflux pumps:*** The efflux mechanism is the primary contributor to antibiotic resistance in mycobacteria. This mechanism expels toxic substances from within cells through cellular membrane proteins known as efflux pumps.

Although efflux is linked to low antibiotic resistance levels in MABC, prolonged exposure to antibiotics at subtherapeutic doses can lead to high resistance levels due to mutations in antibiotic targets^85^. Efflux pumps can be specific to a single substrate or transport various compounds, including antibiotics from different therapeutic classes. The transportation of multiple compounds is associated with the emergence of multidrug resistance^86^.

Efflux in mycobacteria is an intrinsic mechanism of antibiotic resistance; however, limited research exists on the efflux pumps in MABC^84^ ^,^ ^87^ ^,^ ^88^. High antibiotic resistance levels in MABC can be acquired through efflux mechanisms, particularly those involving mycobacterial membrane protein large (*MmpL*)^89^, a member of the resistance-nodulation-division transporter family, and multidrug-resistant efflux pumps, which facilitate the transport of various compounds^90^.

MABC also harbors efflux pumps from the major facilitator superfamily (MFS), which export various substrates. Among these, orthologs of efflux pumps encoded by *M. avium* ^85^ and the MFS efflux pump Tap^91^ have been identified in other mycobacteria, including *M. tuberculosis*, *M. bovis*, and *M. fortuitum* ^92^ ^,^ ^93^. Additionally, efflux pumps of the ATP-binding cassette family, which are responsible for antibiotic efflux^86^, are encoded by MABC^94^.

***Resistance to biocides:*** MABC members exhibit high resistance and tolerance to various biocides (Table 2), including glutaraldehyde^95^ ^-^ ^100^, o-phthalaldehyde^101^, peracetic acid^98^ ^,^ ^102^, quaternary ammonium compounds^98^ ^,^ ^103^ ^-^ ^105^, povidone-iodine^106^, and chlorhexidine^107^ ^,^ ^108^. 

**TABLE 2:** Biocides mechanism of action.

Chemical compound	Mechanism of action	Reference
Glutaraldehyde	Alteration of RNA, DNA, and protein synthesis.	^100^
Ortho-phthalaldehyde	Interaction with amino acids and proteins.	^101^
Peracetic acid	Oxidation and denaturation of proteins and lipids, leading to cellular membrane disorganization.	^102^
Quaternary ammonium compounds	Alteration of cellular membrane permeability.	^105^
Povidone-iodine	Change in protein and nucleic acid structure and synthesis.	^106^
Chlorhexidine	Destruction of the microbial cell membrane.	^108^

Studies have consistently shown that MABC is resistant to glutaraldehyde even after prolonged exposure^95^ ^-^ ^97^ ^,^ ^99^. An evaluation of endoscope cleaning and disinfection procedures revealed that, while glutaraldehyde reduced the mycobacterial load, some cells remained viable post-treatment^97^. Post-surgical infections associated with inadequate disinfection using 2% glutaraldehyde underscore the need for enhanced sterilization protocols^95^ ^,^ ^96^ ^,^ ^99^. The absence of standardized cleaning methods and quality control of glutaraldehyde solutions contributes to persistent MABC contamination^95^ ^,^ ^96^ ^,^ ^98^ ^,^ ^99^. Given these challenges, alternative biocides like o-phthalaldehyde and peracetic acid have demonstrated superior efficacy. Studies have indicated that o-phthalaldehyde (0.3%) eliminates MABC within 5 min, whereas peracetic acid (0.15%) successfully eradicates *M. abscessus* subsp. *bolletii* from endoscopes^98^.

Quaternary ammonium compounds exhibit limited efficacy, as certain MABC isolates survive up to 24 h of exposure^103^. Similarly, MABC isolates display resistance to povidone-iodine and chlorhexidine. *M. abscessus* subsp. *abscessus* and *M. abscessus* subsp. *massiliense* demonstrate high resistance to chlorhexidine^107^.

Additionally, unconventional disinfection methods including the use of chlorine^109^, acetic acid^110^, and ultraviolet-C light^111^ have been investigated. Research has indicated that MABC is more resistant to chlorine than *M. tuberculosis* ^109^, whereas acetic acid (6%, 30 min)^110^ and ultraviolet-C light (5 min at a distance of 3 m) effectively deactivate MABC^111^. These results emphasize the need for enhanced disinfection approaches to manage MABC-associated infections.

The biocide resistance exhibited by MABC has important practical implications for infection control, outbreak prevention, and healthcare practices. Such resistance compromises the efficacy of widely used disinfectants and antiseptics in healthcare settings. Consequently, these agents promote the persistence of microorganisms on surfaces, medical equipment, and other hospital-related materials, as well as in water systems. Accordingly, the risk of cross-transmission and outbreaks increases, particularly in units that perform invasive procedures or provide care to immunocompromised patients^112^ ^-^ ^116^.

Therefore, it is essential to continuously review and monitor cleaning, disinfection, and sterilization protocols. This process ensures the careful selection of biocides with proven activity against MABC and the use of appropriate concentrations and contact times. Not all routinely used antiseptics are effective against MABC, highlighting the importance of validating antisepsis and disinfection protocols, particularly in hospital environments and during invasive procedures. These findings underscore the need for staff training, rigorous healthcare surveillance, and adoption of alternative or combined strategies to prevent outbreaks^112^ ^-^ ^116^.

## FINAL CONSIDERATIONS

MABC is one of the most pathogenic and rapidly growing mycobacterial species worldwide. It causes severe infections that remain particularly difficult to diagnose and treat, especially in immunocompromised patients and in individuals with underlying lung diseases. Advances in taxonomy have improved species and subspecies discrimination. However, clinically relevant differences in antimicrobial susceptibility among MABC subspecies, together with inducible and constitutive macrolide resistance mediated by *erm*(41), continue to complicate therapeutic decision-making and significantly limit effective treatment options. Slow culture-based methods and limited access to advanced molecular tools have hindered current diagnostic approaches, leading to delays in appropriate therapy. Therapeutic management remains suboptimal because of the intrinsic resistance to multiple antibiotic classes, frequent drug-related toxicity, and need for prolonged multidrug regimens. Increasing evidence of tolerance and resistance to commonly used biocides underline the difficulty of controlling MABC in clinical and environmental settings. These factors reinforce the urgent need to develop and implement rapid and accurate diagnostic techniques, standardized susceptibility testing, and novel therapeutic and preventive strategies. Addressing these challenges is critical for improving patient outcomes and reducing the burden of MABC infections.

## Data Availability

Research data is available upon request.
